# Do Forgiveness Campaign Activities Improve Forgiveness, Mental Health, and Flourishing?

**DOI:** 10.3389/ijph.2024.1605341

**Published:** 2024-03-08

**Authors:** Andrea Ortega Bechara, Zhuo Job Chen, Richard G. Cowden, Everett L. Worthington, Loren Toussaint, Nicole Rodriguez, Hernan Guzman Murillo, Man Yee Ho, Maya B. Mathur, Tyler J. VanderWeele

**Affiliations:** ^1^ Department of Psychology, Universidad del Sinú, Montería, Colombia; ^2^ School of Nursing, University of North Carolina at Charlotte, Charlotte, NC, United States; ^3^ Human Flourishing Program and T. H. Chan School of Public Health, Harvard University, Cambridge, MA, United States; ^4^ Department of Psychology, Virginia Commonwealth University, Richmond, VA, United States; ^5^ Luther College, Decorah, IA, United States; ^6^ Department of Social and Behavioural Sciences, City University of Hong Kong, Kowloon, Hong Kong SAR, China; ^7^ Quantitative Sciences Unit, Stanford University, Stanford, CA, United States

**Keywords:** forgiveness, public health, mental health, flourishing, intervention

## Abstract

**Objectives:** To evaluate the effectiveness of a forgiveness public health intervention at promoting forgiveness, mental health, and flourishing.

**Methods:** Colombian students (*N* = 2,878) at a private, nonreligious university were exposed to a 4-week forgiveness community campaign and were assessed pre- and post-campaign.

**Results:** Forgiveness, mental health, and flourishing outcomes showed improvements after the campaign. On average, participants reported engaging in 7.18 (*SD* = 3.99) of the 16 types of campaign activities. The number of types of campaign activities that participants engaged in evidenced a positive linear association with forgiveness, although some activities were more popular than others and some activities were more strongly associated with increased forgiveness. For depression, anxiety, and flourishing, engaging in more activities was generally associated with greater improvements, but the patterns were less consistent relative to forgiveness.

**Conclusion:** This forgiveness public health intervention effectively promoted forgiveness, mental health, and flourishing. Effective campaigns in diverse communities involve promoting mental and physical health through forgiveness. However, recent conflict may hinder acceptance, necessitating political capital for leadership advocating forgiveness initiatives.

## Introduction

Scholarly study of forgiveness as a psychological construct has rapidly increased and its potential relevance to public health has recently been discovered. Increasing evidence suggests positive associations between forgiveness and a wide range of physical and mental health outcomes [1, 2]. A meta-analysis (*N* = 2,323) found that forgiveness interventions were not only related to increases in forgiveness, but also to increases in hope and decreases in anxiety and depression symptoms [3]. Theoretically, symptoms of anxiety and depression are thought to decline because forgiveness provides a sort of closure that forestalls rumination (which is highly involved in both anxiety and depression) [4]. Those improvements in symptoms and the improvements in some relationships that attend forgiveness affect increases in hope [5, 6]. Besides affecting forgiveness, mental health symptoms, and hope, some population-based research has found that flourishing is tied to forgiveness [7, 8]; thus, a reasonable hypothesis is that a forgiveness intervention will also promote increased flourishing.

Most interventions aimed at promoting forgiveness have been done at small scale (e.g., psychoeducational groups, group therapy, individual psychotherapy, do-it-yourself workbooks, or couple therapy) and are labor- and time-intensive [3]. Forgiveness might be understood as a process involving two related dimensions, a decision to treat the offender as a more valued and valuable person (decisional forgiveness) and replacement of negative unforgiving emotions with positive other oriented emotions like empathy, sympathy, compassion, and love (emotional forgiveness) [9]. In Colombia, forgiveness might be needed as a result of transgressions encountered during 60 years of civil conflict [10] plus more common transgressions [11]. Evidence on the effects of large-scale interventions on forgiveness and mental health is lacking. A community-wide forgiveness campaign aimed at individuals through large community-based interventions can increase population-level forgiveness and psychological wellbeing while being cost-effective. Community forgiveness interventions may thus be important for population mental health promotion [12].

### Existing Forgiveness Community Interventions

Three community-wide forgiveness intervention studies have been published. All have been on Christian campuses. Lampton et al. [13] delivered a campus-wide awareness-raising forgiveness campaign at John Brown University (1,100 undergraduates). A convenience sample of people exposed to the campaign (*n* = 23) were compared to people who experienced the campaign plus a 6-h REACH Forgiveness [14] psychoeducational intervention (*n* = 42). People in the awareness-raising only condition improved pre-post on some forgiveness measures (see Table 1).

**TABLE 1:** Means (and standard deviations) for four studies of forgiveness campaigns, including the present study (Monteria, Colombia. 2021).

Outcome	Lampton et al. [13]		Stratton et al. [15]		Griffin et al. [16]		Present study		
	Pre (*n* = 23)	Post (*n* = 23)	Pre (*n* = 29)	Post 2 (*n* = 29)	Wave A (*n* = 881)	Wave D (*n* = 679)	Pre (*n* = 2,878)	Post (*n* = 2,878)	Effect size
Trait forgivingness^a^	35.1 (6.11)	37.1 (5.24)			36.8 (6.30)	37.4 (6.80)	35.9 (7.20)	38.0 (7.10)	0.31
Avoidance^b^	20.8 (6.99)	19.9 (7.19)	19.66 (8.95)	17.90 (8.16)					
Revenge^c^	8.13 (3.44)	7.13 (2.32)	7.41 (2.98)	7.03 (3.21)					
Conciliation^d^			23.22 (5.75)	22.78 (5.62)					
TRIM Total^e^							37.1 (8.80)	39.3 (8.40)	0.24
Positive responses to offender^f^	16.1 (3.92)	19.6 (5.07)	18.11 (5.69)	19.74 (5.40)					
Forgive school friends^g^					4.09 (0.78)	4.12 (0.78)			
Forgive roommate^g^					4.10 (0.92)	4.09 (0.95)			
Forgive teachers^g^					3.23 (0.96)	3.40 (0.90)			
Forgive parents^g^					4.45 (0.80)	4.51 (0.77)			
Knowledge test^h^							0.35 (0.15)	0.67 (0.30)	1.07
Decisional forgiveness^i^							4.02 (0.93)	4.42 (0.75)	0.42
Emotional forgiveness^j^							3.51 (0.85)	3.83 (0.80)	0.40
Forbearance^k^							3.94 (0.86)	4.36 (0.89)	0.45
Depression symptoms^l^							1.37 (1.03)	1.18 (1.05)	−0.18
Anxiety symptoms^m^							1.24 (1.03)	1.14 (1.10)	−0.10
Flourishing^n^							7.60 (1.80)	8.01 (1.75)	0.24

Note. Effect size for the present study is computed as (posttest—pretest)/pooled SD. All pre-post differences are significant at *p* < 0.001. Testing of pre-post differences used multilevel modeling regression with time of assessment at level 1 and individual at level 2. In each regression equation, the outcome variable was regressed on covariates and time (t2 vs. t1). Covariates included age (continuous), sex (female, male), income (lower, higher), marital status (unmarried, married or in a relationship), religious status (not religious, religious), frequency of religious service attendance (continuous), and war survivor status (not a survivor of war, survivor of war). Outcomes measures as follows:

^a^ Trait Forgivingness Scale (range: 10–50).

^b^ Transgression-Related Interpersonal Motivations Inventory-Avoidance subscale (range: 7–35).

^c^ Transgression-Related Interpersonal Motivations Inventory (TRIM)-Revenge subscale (range: 5–25).

^d^ Transgression-Related Interpersonal Motivations Inventory-Conciliation subscale (range: 6–30).

^e^ Sum of Transgression-Related Interpersonal Motivations Inventory subscales of Avoidance (reverse scored), Revenge (reverse scored), and Benevolence (range: 18–90).

^f^ Forgiveness-Positive Responses to the Offender (range: 6–30).

^g^ Forgiveness of school friends, roommate, teachers, and parents are four single-item measures (range: 1–5 each).

^h^ Forgiveness knowledge is expressed as a fraction of correct responses out of 10 (range: 0–1).

^i^ Decision to Forgive Scale (range: 6–30).

^j^ Emotional Forgiveness Scale (range: 8–40).

^k^ Forbearance Scale (range: 1–6).

^l^ Anxiety subscale of the Brief Symptom Index 18 (range: 1–5).

^m^ Depression subscale of the Brief Symptom Index 18 (range: 1–5).

^n^ Flourishing Index (range: 0–10).

Stratton et al. [15] exposed all undergraduate students at Asbury University (1,500 undergraduates) to a community-wide awareness-raising forgiveness intervention. They sampled 114 students, assigning 29 students to an awareness-raising only condition. Other students were assigned either to write a personal essay about forgiveness (*n* = 46), participate in the REACH Forgiveness psychoeducational groups (*n* = 22), or both (*n* = 17). People in the awareness-only campaign improved pre-post (see Table 1).

Both studies revealed potential for using awareness-raising as a public health forgiveness intervention, but the small sample sizes assessed (<0.50% of the student population) were a limitation. Griffin et al. [16] organized a community-wide forgiveness intervention at Luther College (2,500 undergraduates). They targeted the entire student body for assessment up to four times within a semester. The awareness-raising campaign occurred between times three and four. A total of *n* = 1,192 individuals completed one or more assessments. Post-intervention improvements were observed on dispositional forgiveness and feelings of forgiveness, love, and conflict for parents, teachers, friends, and roommates (see Table 1).

Experiences with forgiveness campaigns in the US helped develop methods of engaging community participants. Two lessons emerged. One way to ensure some level of campaign effectiveness is to establish clear goals and to provide five “required” activities (that prior research suggests would produce some effects and were in line with campaign goals).

### Community Forgiveness Intervention Impact and Limitations

The average campaign engagement for each assessed student in Griffin et al. [16] was 2.5 h. Using the dose-response relationship for individual, couple, or group interventions in Wade et al. [3], individuals experiencing a community forgiveness intervention would be predicted to have a 0.25 standard-deviation improvement in forgiveness and a 0.12 standard-deviation decrease in anxiety and depression symptoms if they experienced the average dose of 2.5 h. This estimate relies on the assumption that exposure to public health materials could be compared to the intensity of individual treatments.

Existing community forgiveness interventions possess important limitations. First, most sample sizes are small [13, 15]. Second, all studies involved Christian communities. Christianity emphasizes forgiveness, which could compromise generalizability of the intervention effect. Third, existing studies treated forgiveness-intervention activities as interchangeable. That is, no forgiveness intervention studies have done analyses at the level of activities. The assumption that activities can be equally effective at promoting forgiveness is unsupported empirically. Fourth, existing studies have not established a community-level forgiveness intervention dose-response relationship for forgiveness or mental health outcomes. This is crucial for determining whether future campaigns are cost-effective. Fifth, no effort has been made to determine which individual activities might most effectively produce improvements in forgiveness, mental health, and flourishing.

Practically, many lessons have been learned from existing university community interventions. First, engagement of the administrators, professors, staff, and student body is needed, and that can be engendered by engaging these various stakeholders in planning the campaign. Second, engaging the senior leadership (e.g., university President and Provost) is needed to give weight to the enterprise. Third, raising awareness alone will generally not produce substantial community-wide forgiveness. Activities that induce people to practice forgiveness are necessary. Fourth, campaigns must be tailored to the community. Fifth, clear goals for the campaign must include (at a minimum) the following: (a) an understanding of how forgiveness is defined; (b) information about the benefits of forgiveness to the forgiver; (c) details about where practical forgiveness resources can be found; and (d) practical activities that have evidence supporting their effectiveness.

### The Present Study

We conducted a community forgiveness campaign at Universidad de Sinú, a private, nonreligious university in Monteria, Colombia (student population of 8,987). Changes in forgiveness, mental health, and flourishing were assessed in a large sample (almost three-fold higher than all prior forgiveness campaigns combined). Amount of time spent on, the effectiveness of, and use of each activity were evaluated to help future forgiveness campaign organizers select activities that contribute most to outcomes. This led to four research questions:

1. Was the campaign successful at enhancing forgiveness, mental health, and flourishing?
2. Which types of activities were related to the greatest changes in forgiveness, mental health, and flourishing, controlling for participation in other activities?
3. What was the association between number of types of activities that individuals engaged in and effectiveness in promoting forgiveness, mental health, and flourishing?
4. Which types of activities were most effective at promoting forgiveness, mental health, and flourishing, popular in attracting users, and engaged the most amount of time?

## Methods

### Participants

The sample included 2,878 students in a private, secular university located in Monteria, Colombia. The mean age of the participants was 20.88 years (*SD* = 4.05), most of whom were female (68.80%), lived in a household with at least one minimum wage income (57.75%). More than two-thirds of the sample described themselves as religious (71.43%), somewhat less than national estimates for the Colombian adult population [17]. A minority of the participants were married or in a relationship (17.03%) and identified as a survivor of war (16.17%).

### Design

The study used a pretest-posttest design to evaluate a community forgiveness campaign that included 16 forgiveness-promoting activities delivered over 4 weeks.

### The Campaign for Forgiveness

The campaign aimed at stimulating awareness and experience of forgiveness among university students. Five activities were selected from 35 suggested by Griffin et al. [16] and were adapted for local conditions by student and student affairs leadership, who devised 11 additional activities. The campaign was approved and received partial financial support by the university. The number of participants in each activity was recorded, and respondents self-reported which specific activities they participated in. However, many types of activities could be participated in multiple times. For example, people could attend zero to six webinars on forgiveness, but they reported only that they attended at least one forgiveness webinar. The 16 types of activities are described below.

#### Knowledge-Based Training (2 Activities)

Both activities aimed to build intellectual or experiential knowledge of forgiveness. (a) A 10-item, pre-post, mastery-based Forgiveness Knowledge Test could be taken as often as desired. Respondents with a score of nine or 10 were eligible for a 500,000 pesos (∼$125 USD) lottery. (b) The brief REACH Forgiveness 2-h do-it-yourself workbook to promote experiential knowledge was available (not repeatable).

#### Online Written-Discussion Entries (3 Activities)

(a) Participants could keep a private, confidential online written journal. Daily writing prompts were provided. (b) An online discussion site permitted interaction with anonymous or identifiable participants. (c) A video club forum involved watching and discussing forgiveness video clips.

#### Forgiveness Videos (7 Activities)

Participants could view any or all videos. (a) Three full-length movies had forgiveness themes (*Spiderman 3*, *Invictus*, and *Lessons in Forgiveness*), and any one could be selected. People could participate in an online discussion forum if desired. (b) Videos of four brief expert-talks on forgiveness by Everett Worthington, Fred Luskin, Loren Toussaint, and Robert Enright were available. At least two of the four had to be attended to count as an activity. All four talks earned two activities. (c) Four brief animated films (*Benefits of Forgiveness*, *The Injustice Gap*, *Forgiveness According to Science*, and *What Forgiveness is Not*) were also available to watch and critique. Each counted separately.

#### Webinars (4 Activities)

Experts gave 1-h webinars tailored toward forgiveness. Participants could choose any or all of 20 webinars on the topics of (a) forgiveness (*n* = 6), (b) positive psychology (*n* = 6), (c) yoga (*n* = 4), and (d) mindfulness (*n* = 4). Choosing at least one webinar in any of the four categories counted as an activity.

#### Other Activities

A “forgiveness tree” on campus provided a point for reflection regarding forgiveness, hope, and gratitude, and it could be visited as often as desired. Similarly, at a “forgiveness wall,” people could write about forgiveness, take a photo at the wall, and tag the campaign website. Participants could make a video reciting a forgiveness mantra they created, post it on Instagram, and notify the campaign team of their participation. Participants who were most active on Facebook and Instagram with posts received a small prize.

### Measures

Participants completed the following set of multi-item measures. All psychological assessment measures have strong psychometric properties. Spanish versions of the measures were used where they already existed. When no pre-existing Spanish versions were available, English versions were translated, back-translated, and pilot-tested before use. Alpha estimates of internal consistency across all measures and both time points were ≥0.70.

#### Forgiveness

Ten questions from a four-option multiple-choice Forgiveness Knowledge Test [18] assessed comprehension of ten statements concerning forgiveness (e.g., “Holding onto a sense of injustice has negative effects on … a. physical health, b. relationships, c. neither, d. both”). The 10-item Trait Forgivingness Scale (TFS [19]) assessed the tendency to forgive others across situations and time (e.g., “I am a forgiving person”). The six-item Decision to Forgive Scale (DTFS [20]) measured decisional forgiveness (e.g., “I have decided to forgive him or her”). The eight-item Emotional Forgiveness Scale (EFS [21]) assessed having replaced negative other-oriented emotions with positive ones (e.g., “I care about him/her”). The 18-item Transgression Related Interpersonal Motivations Inventory (TRIM [22]) was used to assess offender-targeted forgiveness motives, including *revenge*, *avoidance*, and *benevolence* (e.g., “I have given up my hurt and resentment”). The nine-item Forbearance Scale [23] was used to assess the tendency to overlook others’ minor transgressions (e.g., “I overlook others’ mistakes”).

#### Mental Health

The six-item anxiety and depression subscales of the Brief Symptom Inventory 18 [24] assessed the degree to which anxiety symptoms (e.g., “Feeling fearful”) or depression symptoms (e.g., “Feeling blue”) had distressed participants in the past 7 days.

#### Flourishing

The 10-item Flourishing Index [25] assessed flourishing in five domains: happiness and life satisfaction, mental and physical health, meaning and purpose, character and virtue, and close social relationships (e.g., “Overall, how satisfied are you with life as a whole these days?” [life satisfaction]; “I understand my purpose in life” [purpose]).

### Procedure

The campaign took place during one semester. Webinar speakers were scheduled. Messages from leaders in the scientific study of forgiveness were pre-recorded. University funding was secured. Animated videos were made. University approval was secured for the forgiveness tree and forgiveness wall. Professors were invited one semester in advance to develop campaign-related course work and projects (some of which were mandatory; others voluntary). Online resources were created and programmed (e.g., knowledge-test scoring and feedback). Advertising began weeks before the campaign. High visibility programming launched the campaign. Activities were voluntarily selected and open to all university students. In a few cases professors made participation in (a choice of) activities mandatory to receive class credit. Post-campaign feedback was provided to administrators and university officials. Of the 2,878 participants, 75.00% participated in assessments as part of a class project, and 25.00% volunteered.

### Analysis

All analyses were performed using R version 4.2. Pre- and post-campaign descriptive statistics were reported for all outcome variables. For research questions one, two, and four, we tested pre-post differences using multilevel modeling regression with time of assessment at level one and individual at level two. In each regression equation, the outcome variable was regressed on covariates and time (t_2_ vs. t_1_), and included random intercepts by participant. Covariates included age (continuous), sex (female, male), income (lower, higher), marital status (unmarried, married or in a relationship), religious status (not religious, religious), frequency of religious service attendance (continuous), and war survivor status (not a survivor of war, survivor of war). Effect sizes for each outcome were computed as (posttest—pretest)/pooled standard deviation. To assess dose-response, linear models were estimated to examine forgiveness, depression, anxiety, and flourishing effects and their association with total number of activities.

## Results

### Research Question 1: Was the Campaign Successful at Enhancing Forgiveness, Mental Health, and Flourishing?

In Table 1, we present pretest and posttest means and standard deviations for each of the nine outcome variables. All pre-post changes were significant (*p*s < 0.001). The effect size for increase in knowledge of forgiveness was *d* = 1.07. The average effect size for improvements in forgiveness outcomes was *d* = 0.36, with more modest effect sizes for depression (*d* = −0.18), anxiety (*d* = −0.10), and flourishing (*d* = 0.24).

### Research Question 2: Which Types of Activities Effected the Greatest Changes in Forgiveness, Mental Health, and Flourishing, Controlling for Engagement in the Other Campaign Activities?

In Table 2, we present coefficients for the unique contribution of each type of activity to pre-post improvements in forgiveness. Using a Bonferroni-corrected alpha (0.0031 for 16 activities), all 16 campaign activities improved knowledge; 13, trait forgivingness and emotional forgiveness; 12, forbearance; 9, forgiveness motivations (i.e., TRIM), and 8, decisional forgiveness. Even though the campaign advertising was not overtly aimed at mental health and flourishing, each was affected. In Table 3 (columns 2–4), we display the effects of each type of campaign activity on mental health outcomes. Of the 16 campaign activities, 16 improved forgiveness; six, depression; four, anxiety; and 12, flourishing.

**TABLE 2:** Effects of types of individual campaign activities on forgiveness outcomes (Monteria, Colombia. 2021).

Campaign activity	Forgiveness outcome: Coefficients B [95% confidence interval]					
	Forgiveness knowledge^a^	State forgiveness^b^	Decisional forgiveness^c^	Emotional forgiveness^d^	Trait forgivingness^e^	Forbearance^f^
Knowledge-based training						
REACH workbook	0.70** [0.62, 0.78]	0.11** [0.04, 0.19]	0.14** [0.05, 0.22]	0.17** [0.09, 0.24]	0.12** [0.05, 0.19]	0.12* [0.03, 0.20]
Knowledge test	1.14** [1.03, 1.26]	0.15* [0.04, 0.26]	0.20** [0.08, 0.33]	0.25** [0.14, 0.36]	0.12* [0.01, 0.22]	0.12* [0.00, 0.24]
Online written entries						
Forgiveness journal	0.48** [0.38, 0.57]	0.19** [0.10, 0.28]	0.21** [0.11, 0.31]	0.22** [0.13, 0.31]	0.19** [0.10, 0.27]	0.18** [0.09, 0.28]
Forgiveness forum	0.48** [0.39, 0.57]	0.15** [0.07, 0.23]	0.12* [0.03, 0.21]	0.18** [0.10, 0.27]	0.15** [0.07, 0.23]	0.16** [0.07, 0.25]
Video club forum	0.48** [0.33, 0.63]	0.12 [-0.01, 0.26]	−0.02 [−0.18, 0.13]	0.20** [0.07, 0.34]	0.13* [0.00, 0.26]	0.23** [0.09, 0.38]
Forgiveness videos						
Video club movies	0.31** [0.22, 0.39]	0.12** [0.05, 0.19]	0.14** [0.05, 0.22]	0.15** [0.07, 0.22]	0.17** [0.10, 0.25]	0.13** [0.05, 0.21]
Expert video	0.25** [0.17, 0.33]	0.10* [0.03, 0.17]	0.09* [0.01, 0.17]	0.17** [0.10, 0.25]	0.16** [0.09, 0.24]	0.19** [0.11, 0.27]
Animated video	0.48** [0.40, 0.57]	0.13** [0.05, 0.20]	0.13** [0.05, 0.22]	0.18** [0.10, 0.25]	0.23** [0.16, 0.31]	0.23** [0.14, 0.31]
Webinars						
Forgiveness	0.40** [0.32, 0.48]	0.18** [0.11, 0.25]	0.13** [0.05, 0.22]	0.20** [0.13, 0.27]	0.20** [0.13, 0.27]	0.21** [0.13, 0.29]
Positive psychology	0.29** [0.21, 0.37]	0.15** [0.08, 0.23]	0.09* [0.00, 0.17]	0.19** [0.11, 0.26]	0.19** [0.12, 0.26]	0.17** [0.09, 0.25]
Yoga	0.17** [0.09, 0.25]	0.07 [−0.01, 0.14]	0.13** [0.04, 0.21]	0.08* [0.01, 0.16]	0.12** [0.05, 0.19]	0.11* [0.02, 0.19]
Mindfulness	0.21** [0.12, 0.29]	0.09* [0.01, 0.17]	0.07 [−0.02, 0.16]	0.15** [0.08, 0.23]	0.19** [0.11, 0.26]	0.14** [0.06, 0.23]
Other activities						
Forgiveness tree	0.21** [0.13, 0.30]	0.08* [0.00, 0.16]	0.08 [−0.00, 0.17]	0.08* [0.01, 0.16]	0.15** [0.08, 0.22]	0.16** [0.07, 0.24]
Forgiveness wall	0.25** [0.17, 0.34]	0.12** [0.04, 0.19]	0.11* [0.02, 0.20]	0.10* [0.02, 0.18]	0.17** [0.09, 0.24]	0.18** [0.10, 0.26]
Mantra	0.18** [0.09, 0.26]	0.13** [0.06, 0.21]	0.14** [0.06, 0.22]	0.16** [0.08, 0.23]	0.17** [0.09, 0.24]	0.17** [0.08, 0.25]
Social media marathon	0.21** [0.13, 0.29]	0.06 [−0.01, 0.14]	0.07 [−0.01, 0.16]	0.11** [0.04, 0.19]	0.09* [0.02, 0.16]	0.09* [0.01, 0.17]

Note. Each cell was a separate multilevel model with time of assessment at level 1 and individual at level 2. In each regression equation, the outcome variable (standardized with *M* = 0 and *SD* = 1) was regressed on covariates, engagement in each of the 16 campaign activities (no, yes), time (t_2_ vs. t_1_), and the cross-level interaction of time with a campaign activity (one campaign activity at a time). Covariates included age (continuous), sex (female, male), income (lower, higher), marital status (unmarried, married or in a relationship), religious status (not religious, religious), frequency of religious service attendance (continuous), and war survivor status (not a survivor of war, survivor of war). Coefficients are interpreted as, holding all covariates and engagement in other campaign activities equal, engaging in the specific campaign activity of interest is associated with B standard deviation increase in the outcome. *p < 0.05, **p < 0.0031 (0.05/16 which is the Bonferroni adjustment for 16 tests on the same outcome).

^a^ Forgiveness Knowledge Test.

^b^ Sum of Transgression-Related Interpersonal Motivations Inventory subscales of Avoidance (reverse scored), Revenge (reverse scored), and Conciliation.

^c^ Decisional Forgiveness Scale.

^d^ Emotional Forgiveness Scale.

^e^ Trait Forgivingness Scale.

^f^ Forbearance Scale.

**TABLE 3:** Effectiveness and prevalence-usage for each of the 16 types of campaign activities (Monteria, Colombia. 2021).

Campaign activity	Outcome: Coefficients B [95% confidence interval]				
	Forgiveness composite	Anxiety symptoms	Depression symptoms	Flourishing	Prevalence of engagement (rank)
Knowledge-based training					
REACH workbook	0.32** [0.24, 0.39] (4)	−0.01 [−0.09, 0.07]	−0.04 [−0.12, 0.03]	0.12** [0.05, 0.19]	**59.35% (4)**
Knowledge test	0.46** [0.36, 0.56] (1)	−0.13* [−**0.24,** −**0.01] (4)**	−0.10 [−0.21, 0.00]	0.21** [0.11, 0.32] (1)	**83.70% (1)**
Online written entries					
Forgiveness journal	0.34** [0.26, 0.42] (2)	−0.05 [−0.14, 0.04]	−0.06 [−0.14, 0.03]	0.16** [0.07, 0.24]	21.02%
Forgiveness forum	0.29** [0.21, 0.37]	0.04 [−0.04, 0.12]	−0.02 [−0.10, 0.06]	0.10* [0.02, 0.18]	26.89%
Video club forum	0.27** [0.14, 0.39]	0.04 [−0.09, 0.18]	0.04 [−0.09, 0.17]	0.12 [−0.01, 0.25]	7.61%
Forgiveness videos					
Video club movies	0.24** [0.17, 0.31]	−0.11* [−0.19, −0.03]	−0.12** [−**0.20,** −**0.05] (5)**	0.16** [0.09, 0.23] (5)	40.86%
Expert video	0.22** [0.15, 0.29]	−0.08* [−0.16, −0.01]	−0.06 [−0.13, 0.01]	0.12** [0.05, 0.20]	45.66%
Animated video	0.32** [0.25, 0.39] (3)	−0.18** [−**0.26,** −**0.10] (1)**	−0.19** [−**0.26,** −**0.11] (1)**	0.18** [0.10, 0.25] (2)	**58.72% (5)**
Webinars					
Forgiveness	0.31** [0.24, 0.38] (5)	−0.16** [−**0.24,** −**0.08] (3)**	−0.16** [−**0.23,** −**0.09] (3)**	0.18** [0.10, 0.25] (2)	51.08%
Positive psychology	0.25** [0.18, 0.32]	−0.18** [−**0.25,** −**0.10] (2)**	−0.18** [−**0.26,** −**0.11] (2)**	0.17** [0.10, 0.24] (4)	43.75%
Yoga	0.15** [0.08, 0.22]	−0.09* [−0.17, −0.01]	−0.09* [−0.17, −0.02]	0.12** [0.05, 0.19]	37.87%
Mindfulness	0.20** [0.13, 0.27]	−0.12** [−**0.20,** −**0.04] (5)**	−0.13** [−**0.20,** −**0.05] (4)**	0.13** [0.06, 0.21]	30.76%
Other activities					
Forgiveness tree	0.17** [0.10, 0.25]	−0.03 [−0.11, 0.05]	−0.06 [−0.13, 0.02]	0.13** [0.06, 0.21]	**59.94% (3)**
Forgiveness wall	0.21** [0.14, 0.29]	−0.08* [−0.16, 0.00]	−0.08* [−0.16, −0.01]	0.06 [−0.01, 0.14]	**60.39% (2)**
Mantra	0.22** [0.15, 0.29]	−0.06 [−0.14, 0.02]	−0.11** [−0.19, −0.04]	0.11** [0.04, 0.18]	41.14%
Social media marathon	0.15** [0.08, 0.22]	−0.02 [−0.09, 0.06]	0.00 [−0.08, 0.07]	0.03 [−0.04, 0.10]	49.76%

Note. Values in parentheses are the rank of each campaign activity within each column, and the top five ranked campaign activities in each column are in bold. For the forgiveness composite (computed as the average of six standardized forgiveness outcomes), two mental health outcomes (depression, anxiety), and flourishing, each cell was a separate multilevel modeling regression with time of assessment at level 1 and individual at level 2. In each regression equation, the outcome variable (standardized with *M* = 0 and *SD* = 1) was regressed on covariates, engagement in each of the 16 campaign activities (no, yes), time (t_2_ vs. t_1_), and the cross-level interaction of time with a campaign activity (one campaign activity at a time). Covariates included age (continuous), sex (female, male), income (lower, higher), marital status (unmarried, married or in a relationship), religious status (not religious, religious), frequency of religious service attendance (continuous), and war survivor status (not a survivor of war, survivor of war). Coefficients are interpreted as, holding all covariates and engagement in other campaign activities equal, engaging in the specific campaign activity of interest is associated with B standard deviation increase in the outcome. *p < 0.05, **p < 0.0031 (0.05/16 which is the Bonferroni adjustment for 16 tests on the same outcome).

### Research Question 3: What Was the Association of Number of Types of Activities in Which Participants Participated and Effectiveness in Forgiveness, Mental Health, and Flourishing?

**FIGURE 1:**
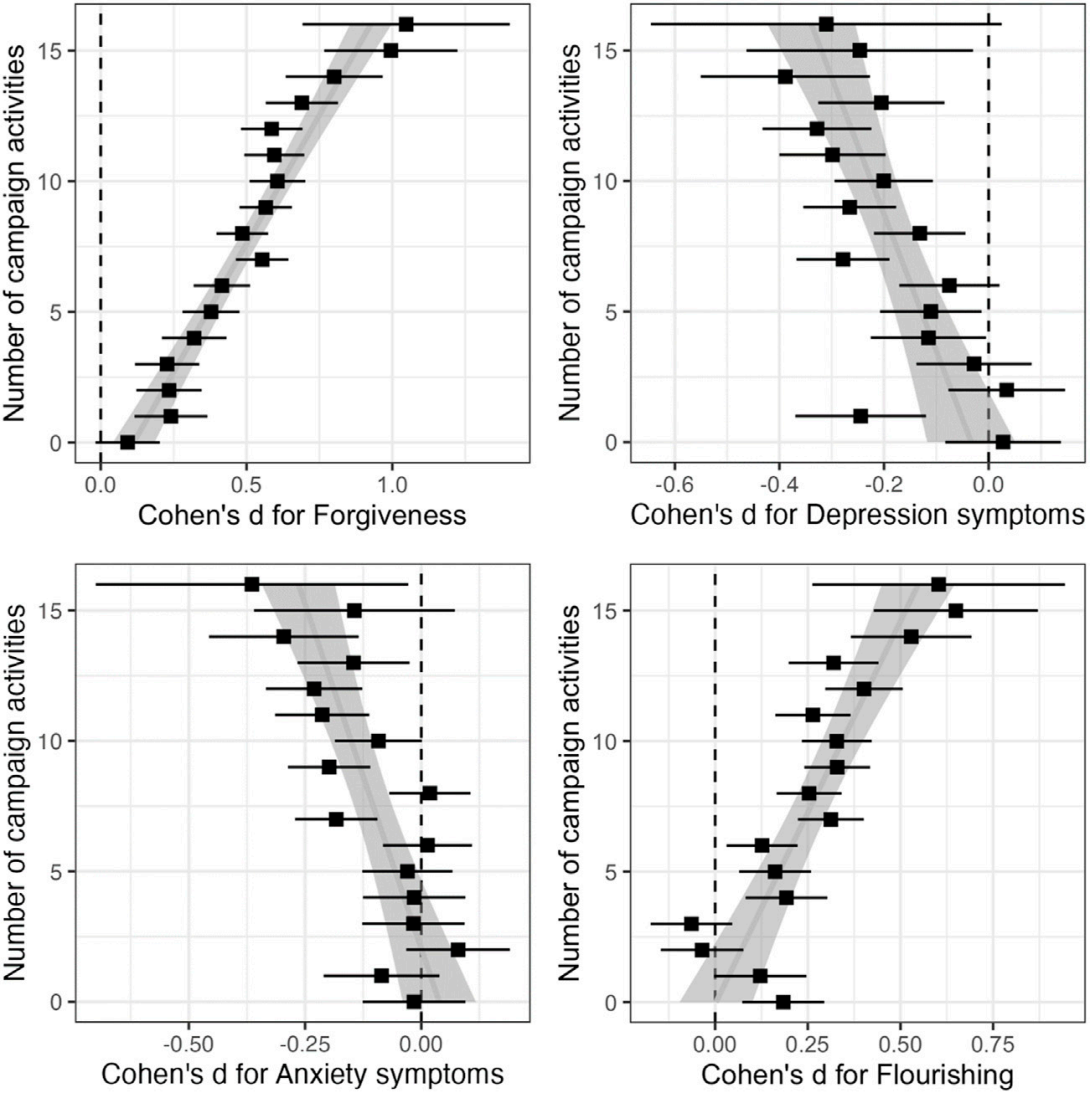
Pre-post effect sizes for engagement in 16 types of campaign activities across forgiveness, mental health, and flourishing outcomes (Monteria, Colombia. 2021). Note. The estimates and confidence intervals for each number of campaign activities are derived from the subset of participants who did exactly that number of activities. The effect size for forgiveness is the average effect size over those of six forgiveness outcomes. The vertical dashed line is drawn at 0 effect size.

Nearly everyone (94.30%) participated in at least one of the 16 activities, and on average people participated in 7.18 (*SD* = 3.99) types of activities. In Figure 1 (top left panel), we observe the approximately linear association between number of types of activities participated in and changes on forgiveness. Engaging in fewer than five types of campaign activities indicated little benefit for forgiveness. The effects of number of activities on mental health (depression symptoms in top right panel, and anxiety symptoms in bottom left panel) and flourishing (bottom right panel) revealed a linear increasing pattern (see the best-fit linear lines). However, there were more deviations from the strict linear pattern with those outcome measures relative to forgiveness.

### Research Question 4: Which Types of Activities Were Most Effective at Promoting Forgiveness, Mental Health, and Flourishing, Popular in Attracting Users, and Engaged the Most Amount of Time?

In Table 3, we provide effectiveness and popularity-usage statistics. The five types of activities associated with the largest changes, ranked by their effect across forgiveness composite (i.e., the mean across the six standardized forgiveness outcomes), were the knowledge test, forgiveness journal, animated video, REACH Forgiveness workbook, and forgiveness webinar. The five most popular programs, based on the frequency of participation, were the knowledge test, forgiveness wall, forgiveness tree, REACH Forgiveness workbook, and animated videos.

In terms of changes in mental health symptoms, the animated videos were associated with the largest reduction in anxiety and depression symptoms; positive psychology webinars were ranked second in effectiveness. For flourishing, the knowledge test, animated videos, and forgiveness webinars produced the largest changes.

Written activities (e.g., forgiveness journal, forgiveness forum and video forum) were not associated with large changes and not frequently selected by participants. Other activities (e.g., forgiveness wall, tree, and mantra) were popular but did not produce large changes. Videos (especially animated videos) were both popular and had large changes. The knowledge-based activities (e.g., the knowledge test and REACH Forgiveness workbook) were frequently used and indicated changes. Forgiveness webinars indicated changes in forgiveness and flourishing, and the positive psychology and mindfulness webinars indicated changes in mental health symptoms and flourishing.

## Discussion

Community campaigns to promote forgiveness can be considered public health interventions [12, 16, 26]. They have the potential to change people’s relational, psychological, and physical health [27]. We investigated the effectiveness of a 4-week forgiveness campaign at increasing forgiveness at a Colombian private, non-religious university. Although it is difficult to validly compare studies with widely different settings, populations, and measures, but we tentatively examined existing studies. A previous 2-week campaign at a Christian college in the US produced average engagement of 2.5 h and a small effect (*d* = 0.06) [16]. In the present study, we used more active than passive activities, studied secular rather than Christian college, tracked individual activities rather than estimated total hours, and assessed mental health and flourishing besides forgiveness.

### Effectiveness of the Campaign as a Whole

The current campaign was considerably more effective in promoting knowledge (*d* = 1.07) and experiential knowledge of forgiveness (*d* = 0.36) and mental health (*d* = 0.17) than was the Luther College campaign (*d* = 0.06) [16]. The large improvement compared to previous work is likely due to a combination of factors, including more intensive efforts to members of the community in participating. For example, more than 1,700 participants completed the brief REACH Forgiveness workbook but at Luther College, only 67 participated in a REACH Forgiveness group. Other activities that required time and thoughtfulness were not at Luther College. Although as a public health intervention, the present campaign allowed choices among many types of activities, it yielded stronger effect sizes on forgiveness than many intensive studies included in a meta-analysis [3] of intensive clinical and psychoeducational forgiveness interventions. Wade et al. [3] meta-analyzed 53 forgiveness studies with an average 44 participants per study and a total of 2,323 participants. The average effect size was 0.56. The current campaign’s effect size was 0.36. That is at the 66th percentile among the 53 individual intensive interventions meta-analyzed. Furthermore, it reached 2,878 participants in a single study—nearly 20% more people than all intervention studies meta-analyzed combined.

The power and reach of this public health campaign to promote forgiveness owes to the buy-in of the entire university community. That includes participation of students, staff, faculty, and administrators, financial support by the university, and engagement of faculty to work activities into their courses and learn enough about forgiveness to lecture on it.

### Participation in the 16 Campaign Activities

There was a linear relationship between number of types of activities engaged in and forgiveness across the range of 16 activities; however, people who participated in four or fewer types of activities experienced little real forgiveness. This is consistent with Worthington’s [26] exposure theory of forgiveness, which argues that the amount of exposure to activities inviting people to forgive is directly related to the amount of forgiveness experienced. The effect of number of types of activities on mental health and flourishing outcomes was generally increasing monotonically, though less definitive. An important caveat is necessary. We were unable to determine number of events people participated in within each “activity.” For example, people could take the knowledge test multiple times, attend one to six forgiveness or positive psychology webinars, and participate in most activities numerous times. Prior theorizing suggests that time spent trying to forgive is causal [26]. In the present study, exposure to different types of activities was confounded with exposure duration.

The most helpful (at forgiving) and popular activities were first, a mastery-based formative knowledge test; second, the REACH Forgiveness workbook (which was non-repeatable); and third, an animated series of videos. Expert forgiveness talks were sixth in both usage and effectiveness. Both the knowledge test and the brief REACH Forgiveness workbook offered formative assessment which may have contributed to their effectiveness. Journaling indicated changes in forgiveness, but not for other outcomes, and was not often chosen. The forgiveness wall and tree were popular, but not effective, possibly because they were largely passive. Generally, the most chosen and effective activities were: (a) brief but not too brief, (b) engaging, (c) focused on learning, motivation, and application, and (d) feedback-providing.

There is a well-established general finding in forgiveness intervention research that time spent trying actively to forgive is linearly related to forgiveness and reduction in depression and anxiety. But for mental health variables, the effect size is about half [3]. Our data were not entirely consistent with this general finding reported in prior work, but it was similar. The Wade et al. [3] finding is based on randomized controlled studies of different durations. The present is a public health intervention allowing community members to select activities instead of agree to a particular psychoeducational intervention. In this community intervention, activities that take longer tended to be more effective (if selected) than shorter activities; however, activities of longer duration need to be incentivized to draw participation from busy students embedded in an open college community.

The most helpful and popular activities for mental health were animated videos, which were created specifically for the present campaign, and positive psychology and forgiveness webinars. The Forgiveness Knowledge Test, forgiveness tree, and brief REACH Forgiveness workbook were somewhat less effective but more frequently used. Generally, the activities that were the most popular and effective were entertaining, motivating, and educating (e.g., animated videos, webinars, knowledge test, and the brief REACH Forgiveness workbook).

Overall, activities that involved writing (with the exception of the knowledge test and the brief REACH Forgiveness workbook) were not popular; even when they were selected, they were generally not very effective at promoting forgiveness, reducing mental health symptoms, or promoting flourishing, consistent with Stratton et al. [15] Nation et al. [27] provided an online REACH Forgiveness do-it-yourself intervention taking about 7 h to complete, and they found that only 26% of those who started the intervention completed it, which is consistent with our findings that people preferred activities taking one to 3 h.

### Limitations

The current study has several strengths but also limitations. First, participants were university students with limited histories of offense. However, about one-fourth of the sample had experienced a war-related event, perhaps unsurprisingly, given Colombia’s history of civil conflict [28]. Second, the ideal research design would have included a control condition, which would have required a closely yoked university in the same region at about the same size and make up of students at Universidad de Sinú. This was practically not possible. Third, participation and activities were not randomized, so the changes in outcomes may not reflect causal effects. Fourth, we did not assess which types of activities were part of course work for credit, how much credit was received, and which activities certain faculty might have made mandatory (while still permitting choices of which specific activities to participate in). Fifth, we did not assess how much time was spent on each activity. Sixth, because of spillover, it is possible that there were effects of the campaign even on those who did not participate in any activities. Seventh, there was extraordinary cooperation by the university, which might limit generalizability of the findings. However, the present study encourages public health interventionists to engage an entire community.

### Public Health Implications

Throughout the world, there is a major need to promote better mental health, research is accumulating to show that, if forgiveness is experienced, mental health and flourishing generally also increase. The findings of the present study suggest that this might also apply to community settings. There is considerable potential for scaling up implementation of forgiveness campaigns to larger communities, perhaps even in towns or smaller cities in which key communities could be mobilized at the same time and that could produce fiscal gains relative to costs [29]. We suggest that perhaps even nation-states might employ a coordinated effort to promote forgiveness in mediating structures [30], such as universities, secondary schools, workplaces, and public community organizations (e.g., living units or libraries).

The implications of the present study for future forgiveness campaigns are numerous. The key points include: (a) selecting an appropriate size of community and perhaps testing the method in coordinated communities simultaneously; (b) using lessons from this study to promote engagement and support of community and leaders; (c) selection of number, diversity, and dissemination of campaign activities to include the five goal-relevant activities plus others that fit with and engage the specific community; (d) ensuring the community has the capacity for monitoring, evaluation, and learning; and (e) selecting about 14 types of activities that are engaging and long enough to make an impact. Securing buy-in from key community leaders, administrators, and power-brokers within the community is crucial to campaign success.

Practical and policy implications for constructing future effective campaigns for deployment in communities besides universities include the desirability of improving societal flourishing by promoting forgiveness. Yet benefits to forgiving and flourishing are balanced by the recognition that some societies with a recent history of conflict and turmoil might resist a public health initiative to promote forgiveness, especially if it is too soon after the end of conflict. Also, advocating forgiveness will likely require political capital of leadership. In a nation of diverse and often antagonistic beliefs and values, there will certainly be opposition to such a policy. Nonetheless, public health benefits are potentially worthwhile.
